# Pro-Inflammatory Macrophage Phenotype Skewing Induced by Tumor Treating Fields (TTFields)

**DOI:** 10.3390/ijms262412086

**Published:** 2025-12-16

**Authors:** Tal Kan, Yiftah Barsheshet, Tharwat Haj Khalil, Boris Brant, Tali Voloshin, Kerem Ben-Meir, Simona Zisman Rosen, Moshe Giladi, Uri Weinberg, Yoram Palti

**Affiliations:** Novocure Ltd., Haifa 3190500, Israel; tkan@novocure.com (T.K.); ybarsheshet@novocure.com (Y.B.); thajkhalil@novocure.com (T.H.K.); bbrant@novocure.com (B.B.); kben-meir@novocure.com (K.B.-M.); srosen@novocure.com (S.Z.R.); weinberg@novocure.com (U.W.); yoram@novocure.com (Y.P.)

**Keywords:** Tumor Treating Fields (TTFields), macrophage polarization, immunomodulation, non-small cell lung cancer (NSCLC), GEF-H1, NF-κB, MyD88

## Abstract

Tumor-associated macrophages (TAMs) are abundant in the tumor microenvironment (TME) and often adopt an M2-like immunosuppressive phenotype that promotes tumor growth. Reprogramming TAMs toward an M1-like pro-inflammatory state is an attractive therapeutic strategy. Tumor Treating Fields (TTFields), an FDA-approved, electric-field–based therapy, has recently been suggested to modulate immune responses in addition to its established anti-mitotic activity. Here, we investigated the direct effects of TTFields on macrophage activation and function. Murine bone marrow–derived macrophages (BMDMs) were polarized toward a pro-inflammatory M1-like phenotype or an anti-inflammatory M2-like phenotype and exposed to TTFields. TTFields rapidly activated guanine nucleotide exchange factor-H1 (GEF-H1), and downstream nuclear factor kappa B (NF-κB) and activator protein-1 (AP-1, via c-Jun N-terminal kinase [JNK]) signaling. Functionally, TTFields reprogrammed M2-like macrophages by increasing major histocompatibility complex class II (MHC-II) and cluster of differentiation 80 (CD80); reducing arginase-1 (Arg1); and elevating secretion of chemokine (C-X-C motif) ligand 1 (CXCL1), interleukin-6 (IL-6), IL-1β, and IL-12 subunit p70 (IL-12p70). In interferon gamma (IFN-γ)-primed macrophages, TTFields provided a secondary signal, driving myeloid differentiation primary response 88 (MyD88)-dependent expression of inducible nitric oxide synthase (iNOS). In vivo, TTFields reduced tumor burden in an orthotopic murine lung cancer model and increased iNOS expression in both M1-like and a subset of M2-like TAMs. These findings demonstrate that TTFields directly reprogram macrophages toward a pro-inflammatory phenotype, suggesting a novel immunomodulatory mechanism that may enhance anti-tumor immunity in the TME.

## 1. Introduction

Tumor-associated macrophages (TAMs) are abundant infiltrating immune cells in most solid tumors and play pivotal roles in cancer progression and immune evasion [1]. TAMs exhibit remarkable phenotypic plasticity, but they are often skewed toward an M2-like (alternatively activated) state in the tumor milieu, which promotes tumor growth, angiogenesis, and immunosuppression [2,3]. These M2-polarized TAMs secrete anti-inflammatory and pro-tumoral factors (e.g., interleukin (IL)-10, transforming growth factor (TGF-β), vascular endothelial growth factor) and inhibit effective anti-tumor immunity, correlating with poor patient outcomes [4,5,6,7]. By contrast, M1-polarized (classically activated) macrophages produce pro-inflammatory mediators (e.g., nitric oxide via inducible nitric oxide synthase (iNOS), tumor necrosis factor (TNF)-α, IL-12) and can attack tumor cells and stimulate adaptive immunity [3]. An elevated ratio of M1 to M2 TAMs in the tumor microenvironment is associated with improved clinical outcomes across multiple cancers [8]. Given their central role in orchestrating the immune microenvironment, TAMs have emerged as a promising therapeutic target: strategies include depleting TAMs, blocking their recruitment, or reprogramming TAMs from an M2- to an M1-like phenotype to restore anti-tumor immunity [8,9]. Re-educating TAMs toward a pro-inflammatory state has been shown to enhance tumor control in preclinical models and is a key goal for cancer immunotherapy.

Tumor Treating Fields (TTFields) are an established, FDA-approved treatment modality for glioblastoma (GBM), malignant pleural mesothelioma, and metastatic non-small-cell lung cancer (NSCLC), using alternating low-intensity, intermediate-frequency electric fields to selectively disrupt cancer cell division [10]. The specific frequency for treatment of each tumor type is empirically identified [11]. TTFields cause misalignment of polar macromolecules like tubulin and septin, perturbing mitotic spindle assembly and cytokinesis in tumor cells [11,12]. Dielectrophoretic forces generated by TTFields converge at the cleavage furrow during telophase, causing organelle mislocalization and membrane blebbing [13]. These antimitotic effects lead to tumor cell death [11,14,15].

Beyond the direct cytotoxic effects on tumor cells, emerging evidence suggests that TTFields can also modulate the tumor immune microenvironment [16,17,18]. Tumor cells killed by TTFields undergo forms of immunogenic cell death (ICD) that release danger-associated molecular patterns (DAMPs) such as HMGB1 (high mobility group box) and ATP (adenosine triphosphate), thereby promoting dendritic cell maturation and T cell activation [16,17]. Preclinical studies and clinical data indicate that TTFields therapy may increase tumor-infiltrating lymphocytes and synergize with immune checkpoint inhibitors (ICIs) [16,17,19,20,21]. Recent findings also suggest that TTFields may induce leakage of genomic DNA into the cytosol, potentially activating the cGAS-STING (the cytosolic DNA sensor cyclic GMP-AMP synthase; STimulator of INterferon Genes) and AIM2 (absent in melanoma 2) inflammasome pathways within tumor cells [18]. This activation can enhance type I interferon signaling and proinflammatory cytokine release, ultimately supporting T cell priming and clonal expansion [18]. These findings established that TTFields can act as a physical stimulus capable of engaging innate immune sensors in tumor cells, thereby linking the biophysical effects of the therapy to immune activation. However, the mechanisms by which TTFields may directly influence innate immune cells, including macrophages, remain incompletely understood.

Recent insights into innate immunity have revealed that disruption of cytoskeletal dynamics can act as an intrinsic danger signal [22]. GEF-H1 (also known as ARHGEF2), a microtubule-associated Rho guanine nucleotide exchange factor, serves as a key molecular bridge linking microtubule stability to RhoA activation and actin cytoskeleton remodeling. Upon microtubule destabilization, GEF-H1 is released and activated, leading to the stimulation of RhoA/ROCK signaling and downstream pathways such as NF-κB and AP-1, which drive pro-inflammatory gene expression [22,23]. Notably, Kashyap et al. demonstrated that microtubule-targeting chemotherapies rely on GEF-H1 signaling to activate dendritic cells and promote anti-tumor immune responses [22]. The GEF-H1-RhoA-ROCK axis also orchestrates actin remodeling and macrophage behavior, regulating migration, phagocytosis, and contractility. Activation of this pathway induces actomyosin contractility, stress fiber formation, and increased cellular tension, hallmarks of M1-like pro-inflammatory macrophages, while inhibition of RhoA or ROCK promotes M2-like elongated morphologies and anti-inflammatory gene expression [19,20]. Thus, dynamic actin cytoskeletal remodeling, driven by coordinated actin polymerization and myosin-dependent contractility, underlies macrophage plasticity and integrates microtubule-derived mechanical cues into immune cell activation [24]. In tumor cells, TTFields have been shown to activate the GEF-H1 signaling axis, which in turn triggers RhoA/ROCK pathway activation [25]. Given that TTFields disrupt microtubules in tumor cells, we hypothesized that TTFields might similarly perturb the cytoskeleton of macrophages and activate GEF-H1–mediated innate immune signaling, thereby skewing macrophages toward a pro-inflammatory (M1) phenotype.

In this study, we investigated the direct impact of TTFields on macrophage polarization, activation, and signaling. Using an in vitro model of murine bone marrow-derived macrophages (BMDMs), we examined whether TTFields exposure could convert M2-polarized macrophages toward an M1-like state and/or enhance the activation of classically polarized M1 macrophages. We assessed changes in surface markers, cytokine secretion profiles, and activation of key signaling molecules in macrophages treated with TTFields. Our findings reveal that TTFields treatment drives macrophages to acquire M1 characteristics and activates innate immune pathways associated with macrophage pro-inflammatory polarization. These results shed light on a novel immunomodulatory mechanism of TTFields and suggest that TTFields therapy, in addition to its direct tumoricidal effects, may potentiate anti-tumor immunity by reprogramming TAMs in the tumor microenvironment.

## 2. Results

### 2.1. TTFields Activate GEF-H1 and NF-κB Signaling in Macrophages

**Figure 1:**
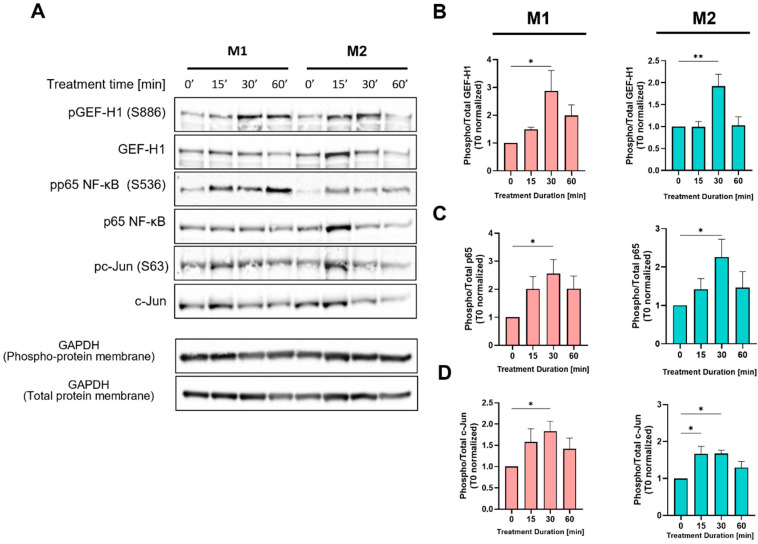
TTFields activate GEF-H1 and drive macrophage transcriptional reprogramming via c-Jun and p65. BMDMs were polarized to either an M1 state (IFN-γ + LPS) or an M2 state (IL-4) and then exposed to TTFields. (**A**,**B**) Western blot analysis of phosphorylated GEF-H1 (Ser886) following TTFields treatment at 15, 30, and 60 min in M1 and M2 macrophages. (**A**,**C**) Phosphorylation of NF-κB p65 (Ser536) in BMDMs treated with TTFields. (**A**,**D**) Phosphorylation of c-Jun (Ser63) in TTFields-treated macrophages. Densitometric analysis for phosphorylation fold change is shown as mean ± SEM; N ≥ 3. * *p* < 0.05, and ** *p* < 0.01; one-way ANOVA followed by Dunnett test compared with time zero.

We first investigated intracellular signaling events potentially triggered by TTFields, aiming to identify mechanisms that may drive subsequent phenotypic changes in macrophages. Since TTFields are known to disrupt microtubule dynamics, we specifically examined GEF-H1, a well-established sensor of microtubule integrity. The GEF-H1/RhoA/ROCK pathway has previously been demonstrated to be activated by TTFields in cancer cells and functions as an upstream regulator of NF-κB in myeloid cells [22,25]. BMDMs were polarized to either an M1 state (IFN-γ and LPS) or an M2 state (IL-4) and then treated with TTFields (at 150 kHz, the frequency used for treating NSCLC) [26]. GEF-H1 activation (p-Ser886) was assessed in BMDMs following TTFields application for 15, 30, and 60 min. Western blot assays demonstrated that TTFields rapidly trigger this pathway in both M1 and M2 polarized macrophages (Figure 1A,B). One major downstream consequence of GEF-H1/RhoA/ROCK pathway activation in immune cells is the stimulation of NF-κB and AP-1 transcription factors that drive pro-inflammatory gene expression [22]. We found that TTFields can activate NF-κB p65 in macrophages (Figure 1A), without detectable STING pathway activation. Figure 1C shows that the phosphorylation of NF-κB p65 (on Ser536, indicative of activation and nuclear translocation) increased in TTFields-treated macrophages, peaking around 30 min into treatment. In addition to NF-κB, TTFields also engaged the c-Jun N-terminal kinase (JNK)/AP-1 pathway: phosphorylated c-Jun levels (a component of AP-1) were increased after TTFields exposure (Figure 1D).

### 2.2. TTFields Promote a Shift of Macrophages Toward a Pro-Inflammatory Phenotype

**Figure 2:**
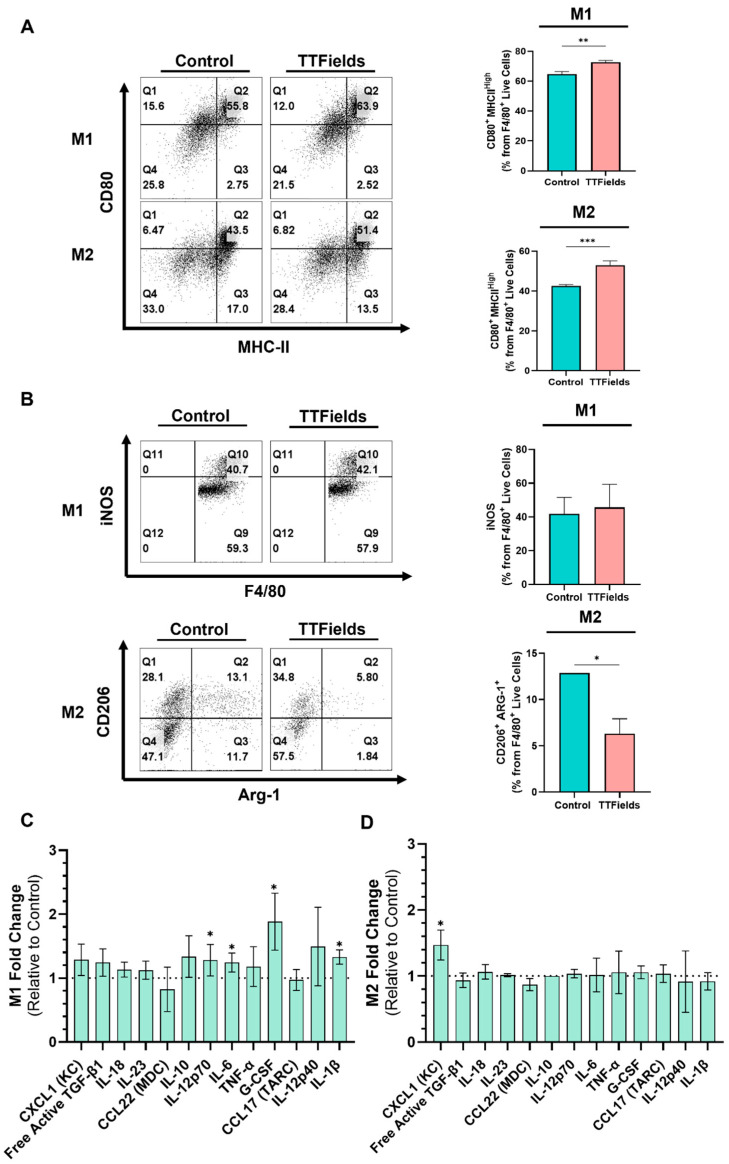
Exposure of polarized BMDMs to TTFields enhanced activation markers and reduced M2-associated markers. BMDMs were polarized to an M1 state (IFN-γ + LPS) or an M2 state (IL-4) and then ex-posed to TTFields. (**A**) Flow cytometric analysis of surface activation markers showing CD80 and MHC-II expression in M1- and M2-polarized macrophages with or without TTFields treatment. (**B**) Intracellular staining for Arg1 and iNOS in polarized macrophages following TTFields exposure. Data represent the percentage of positive cells mean ± SEM; N ≥ 3. * *p* < 0.05, ** *p* < 0.01, and *** *p* < 0.001; Student’s *t*-test. (**C**,**D**) Cytokine and chemokine secretion profile measured by multiplex analysis in supernatants from M1- and M2-polarized BMDMs with or without TTFields treatment. mean ± SEM; N = 3. * *p* < 0.05, ** *p* < 0.01, and *** *p* < 0.001; Student’s *t*-test.

We next examined whether TTFields treatment could modulate the phenotype of polarized macrophages. Flow cytometric analysis revealed that TTFields drove clear changes in macrophage activation markers consistent with M1 polarization (Figure 2). In M2-polarized macrophages, TTFields exposure caused a significant upregulation of the co-stimulatory molecule CD80 and major histocompatibility complex class II (MHC II) compared to untreated M2 cells (Figure 2A). Furthermore, TTFields reduced expression of the M2-associated enzyme Arg1 (Figure 2B). This indicates that TTFields can reprogram M2-polarized macrophages toward an M1-like phenotype. In M1 macrophages, TTFields modestly increased CD80 and MHC II without changing the fraction of iNOS^+^ cells (Figure 2A,B). Taken together, these data demonstrate that TTFields skew macrophage polarization toward a pro-inflammatory, antigen-presenting phenotype.

To assess functional consequences of TTFields-induced macrophage polarization, we measured the secretion of a panel of cytokines and chemokines associated with either M1 or M2 phenotypes. IFN-γ and LPS–stimulated macrophages secreted high levels of classical M1 cytokines (e.g., IL-6, IL-1β, TNF-α, IL-12/p70) and chemokines (e.g., CXCL1) (Figure S1). The multiplex analysis revealed that TTFields exposure broadly augmented the pro-inflammatory secretory profile of macrophages. In M1 macrophages, TTFields further reinforced the inflammatory cytokine output. Notably, IL-12/p70, granulocyte colony-stimulating factor (G-CSF), IL-1β and IL-6 were significantly elevated with TTFields relative to IFN-γ and LPS alone (Figure 2C). These data suggest that TTFields change functionally polarized macrophages to secrete a mix of pro-inflammatory mediators. In M2 macrophages, among the 13 analytes measured, the most notable change was observed in CXCL1 (also known as KC), a chemokine that recruits neutrophils and is typically produced by M1-like myeloid cells [27,28]. Figure S1 illustrates that M2–polarized macrophages had low baseline secretion of CXCL1, but upon TTFields treatment, CXCL1 levels rose significantly (Figure 2C). This indicates that TTFields can trigger M2 macrophages to secrete chemokines associated with inflammation. Interestingly, this marked increase in CXCL1 aligns closely with the activation of the transcription factor c-Jun, known to drive pro-inflammatory gene expression, suggesting a mechanistic link between TTFields-induced intracellular signaling and macrophage cytokine secretion. Importantly, TTFields did not induce a generalized hypersecretion of all cytokines, indicating a skewing specifically toward an M1-associated secretory profile rather than a global activation of macrophages.

We also examined the effect of TTFields on non-polarized (M0) BMDMs and observed only minimal changes in iNOS and activation markers under these conditions, consistent with the notion that naïve macrophages require appropriate priming and a second signal to undergo full M1 differentiation [29]. In line with current concepts that tumor-associated macrophages represent pre-educated, polarized or hybrid states rather than true M0 cells, our study therefore focused on how TTFields modulate IFN-γ-primed and IL-4–polarized macrophages as more relevant surrogates of TAM subsets [3,30,31].

### 2.3. TTFields Act as a “Second Signal” to Induce iNOS in M1 Macrophages via a MyD88-Dependent Pathway

**Figure 3:**
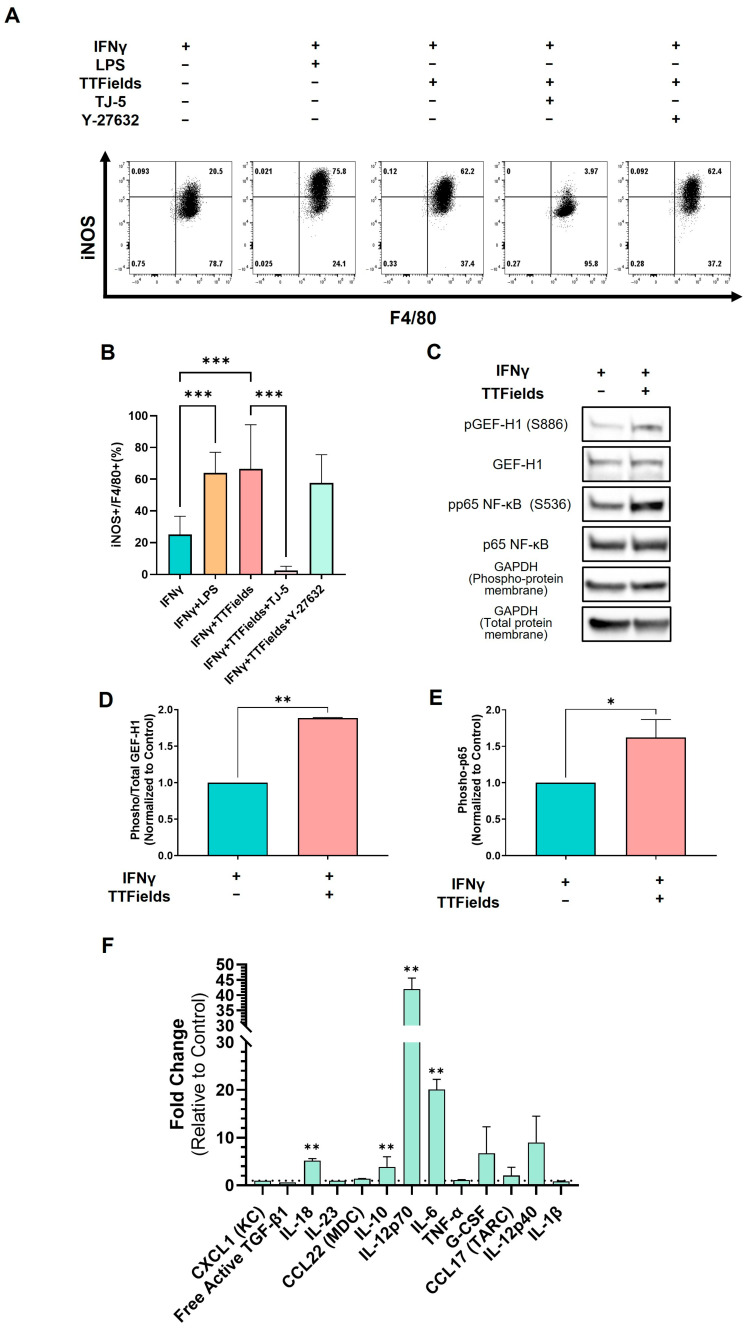
TTFields-induced iNOS expression and cytokine secretion in polarized macrophages. BMDMs were polarized with IFN-γ or IL-4 and then exposed to TTFields with or without pharmacological in-hibitors, or LPS. iNOS protein expression was assessed by flow cytometry. (**A**,**B**) Comparison of iNOS levels in IFN-γ–primed macrophages treated with TTFields or LPS, and the effect of MyD88 inhibition (TJ-M2010-5) or ROCK inhibition (Y-27632). (**C**–**E**) Western blot analysis of GEF-H1 and NF-κB p65 activation in IFN-γ–primed macrophages exposed to TTFields. (**F**) Cytokine and chemokine secretion profile measured by multiplex analysis in supernatants from IFN-γ–primed BMDMs. mean ± SEM; N = 3. * *p* < 0.05, ** *p* < 0.01, and *** *p* < 0.001; Student’s *t*-test.

One hallmark of classically activated M1 macrophages is the expression of iNOS, which produces nitric oxide for pathogen and tumor cell killing. In murine macrophages, robust iNOS induction typically requires two signals: an IFN-γ-mediated priming (signal 1) and an additional inflammatory trigger such as LPS (signal 2) acting through Toll-like receptors [3]. We tested whether TTFields could substitute for LPS as an activation signal for iNOS expression. As expected, BMDMs treated with IFN-γ alone showed minimal iNOS protein expression, whereas IFN-γ with LPS elicited high iNOS levels (Figure 3A,B). Remarkably, IFN-γ and TTFields treatment induced iNOS to a similar extent as the IFN-γ and LPS positive control (Figure 3A,B). This result indicates that TTFields can provide the necessary secondary activation signal to drive iNOS expression in primed macrophages, effectively mimicking LPS-like inflammatory stimulation.

We also investigated the involvement of innate receptor signaling in TTFields-induced macrophage activation. LPS signals through Toll-like receptor 4 and the MyD88 adaptor to activate NF-κB and drive iNOS transcription [32]. We used the MyD88 inhibitor TJ-M2010-5 (TJ-5) to determine if blocking MyD88-dependent pathways would affect TTFields’ ability to induce iNOS. The addition of TJ-5 to IFN-γ-primed macrophages completely abolished iNOS induction by TTFields. By contrast, and unexpectedly, pharmacologic inhibition of ROCK with Y-27632 had no detectable effect (Figure 3A,B). These findings demonstrate that TTFields-triggered iNOS expression requires MyD88-dependent signaling, strongly suggesting that TTFields engage innate inflammatory pathways analogous to Toll-like/IL-1 receptor stimulation. Further analysis confirmed increased activation of both GEF-H1 and NF-κB p65 upon TTFields treatment, paralleling the results obtained in Figure 1 where differentiated macrophages were tested (Figure 3C–E).

To extend these findings, we performed additional multiplex analyses of supernatants from TTFields-treated macrophages (Figure S2). IL-18, IL-12p70, and IL-6 secretion was significantly elevated in IFN-γ-polarized macrophages following TTFields exposure, together with increased levels of IL-10, an anti-inflammatory cytokine (Figure 3F); however, a comparable IL-10 increase was seen in LPS-polarized macrophages, suggesting counter-regulatory feedback rather than an M2-skewing effect [33]. These results indicate that TTFields reshape cytokine output, enhancing pro-inflammatory mediators in IFN-γ–primed macrophages while eliciting a concurrent homeostatic IL-10 response.

In summary, TTFields treatment provides the necessary second signal to fully activate macrophages, and this effect operates through MyD88, implying the involvement of downstream NF-κB/IκB or inflammasome pathways common to innate immune receptor signaling.

### 2.4. Therapeutic Effects of TTFields on Tumor Growth and Macrophage Composition in an In Vivo Lung Cancer Model

**Figure 4:**
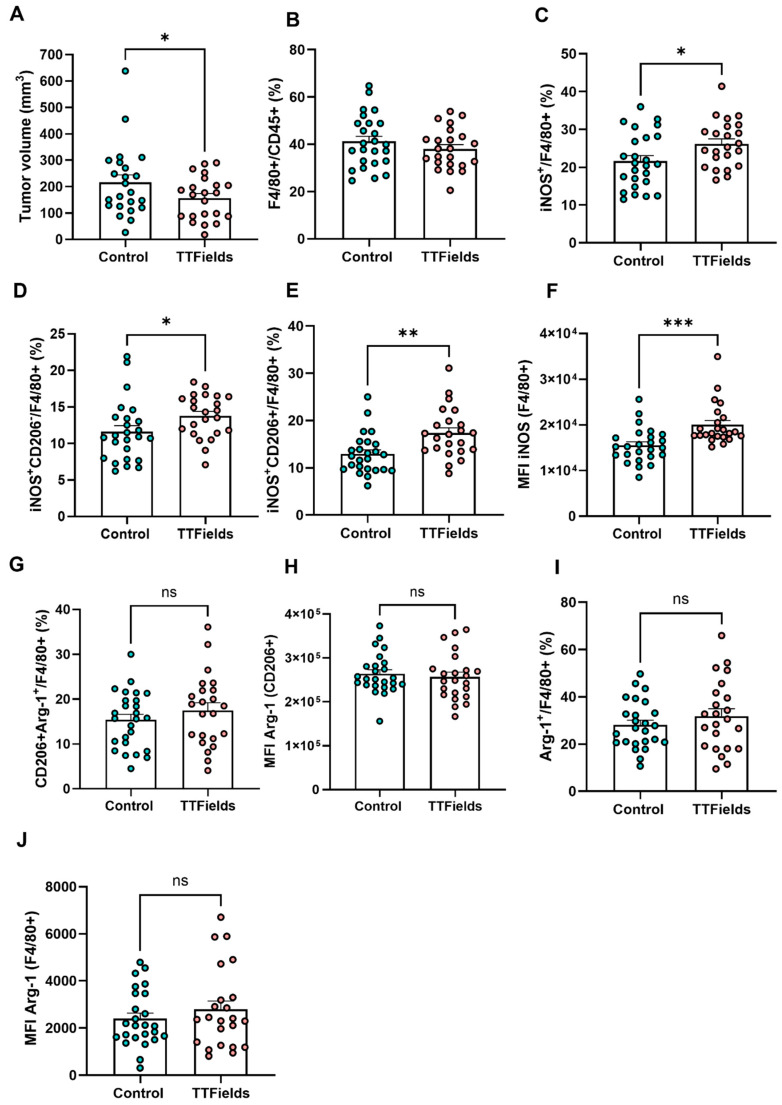
TTFields reduce tumor burden and modulate macrophage iNOS expression in an orthotopic lung cancer model. Orthotopic lung tumors were established and treated with TTFields for ten days before resection and analysis. (**A**) Tumor volume following TTFields or control treatment. (**B**) Proportion of total macrophages (F4/80^+^) among tumor-infiltrating leukocytes. (**C**) Percentage of iNOS^+^ cells within the macrophage compartment. (**D**,**E**) Frequencies of iNOS^+^ cells in CD206^−^ (M1-like) and CD206^+^ (M2-like) macrophages, respectively. (**F**) Median fluorescence intensity (MFI) of iNOS in total macrophages. (**G**,**H**) Frequency and MFI of Arg1^+^ cells in M2-like (CD206^+^/Arg1^+^) macrophages. (**I**,**J**) Arg1 expression in total macrophages, shown as percent positive cells (**I**) and MFI (**J**). n = 22–23 mice per group. Values are mean ± SD. * *p* < 0.05, ** *p* < 0.01, and *** *p* < 0.001; Student’s *t*-test. ns, not significant.

In the next stage, we aimed to investigate the phenomenon in vivo. Following ten days of TTFields application in an orthotopic lung cancer model, tumors were resected and analyzed to assess both tumor burden and macrophage composition within the tumor microenvironment. TTFields treatment led to a significant reduction in tumor volume compared to controls (Figure 4A), without altering the overall proportion of macrophages (F4/80^+^) among tumor-infiltrating leukocytes (Figure 4B). Within the macrophage compartment, TTFields significantly increased the proportion of iNOS^+^ cells (Figure 4C), an effect that was pronounced in both M1-like subset defined as CD206^−^ and M2-like subset (CD206^+^) (Figure 4D–E). MFI analysis revealed higher iNOS expression across the total macrophage population following TTFields (Figure 4F). Notably, unlike our in vitro findings where TTFields reduced Arg1 in M2-polarized macrophages, TTFields did not alter the prevalence of canonical M2-like macrophages (CD206^+^/Arg1^+^) (Figure 4G) or the Arg1 MFI within this subset (Figure 4H). Likewise, Arg1 across the total macrophage population, both the percentage of Arg1^+^ cells (Figure 4I) and Arg1 MFI (Figure 4J), remained unchanged. Together, these findings indicate that TTFields suppress tumor growth while promoting a selective functional reprogramming of tumor-associated macrophages, characterized by enhanced iNOS expression in both M1-like and a subset of M2-like macrophages, without depleting the M2 population or reducing Arg1 levels in vivo.

## 3. Discussion

Our study demonstrates that TTFields have a direct immunomodulatory effect on macrophages, skewing them toward a pro-inflammatory, anti-tumor phenotype. In vitro, TTFields treatment of BMDMs resulted in increased expression of M1-associated markers (MHC II, CD80, iNOS) while attenuating Arg1^+^ M2 macrophages. Specifically, TTFields-treated macrophages secreted higher levels of key pro-inflammatory cytokines and chemokines, as revealed by our cytokine array analysis, confirming their shift toward an M1-like secretory profile. These findings reveal a novel aspect of TTFields’ mechanism of action: reprogramming TAMs to a more immunostimulatory state. All macrophage experiments were performed at 150 kHz, the frequency reported as optimal for NSCLC tumor cells; other frequencies, such as 200 kHz, relevant to glioblastoma, are likewise worthy of evaluation and should be assessed in future studies. Given the central role of TAMs in orchestrating tumor immunity, this TTFields-induced macrophage polarization could help counteract the immunosuppressive milieu in tumors and thereby potentiate anti-tumor immune responses.

In contrast to our in vitro findings, where TTFields exposure of IL-4-polarized M2 macrophages reduced Arg1 and increased MHC-II/CD80 and pro-inflammatory mediators consistent with an M1-like shift, the in vivo data show a different pattern: in the orthotopic lung cancer model, TTFields significantly increase iNOS in both CD206^−^ (M1-like) and CD206^+^ (M2-like) TAMs, but do not reduce Arg1 frequency or MFI in CD206^+^ TAMs or in total macrophages. Thus, in vivo TTFields primarily induce a functional iNOS^+^ shift without fully extinguishing M2-associated Arg1 expression. This apparent discrepancy likely reflects TAM heterogeneity and the existence of hybrid or intermediate states in tumors, where macrophages can co-express classical M1 (e.g., iNOS) and M2 (e.g., Arg1) markers rather than conforming to binary categories. Moreover, Arg1 expression in TAMs is sustained by a complex tumor milieu, including IL-4/IL-13, IL-10, TGF-β, lactic acid, hypoxia, and metabolic crosstalk, which is not fully recapitulated by IL-4 alone in vitro [34,35,36,37]. In this context, the tumor microenvironment naturally promotes mixed iNOS^+^/Arg1^+^ phenotypes, and TTFields appear to overlay an iNOS^+^, pro-inflammatory program onto an Arg1-maintaining background, generating more “hybrid” states rather than fully depleting M2-like TAMs. Future studies using macrophage-tumor cell co-cultures or tumor-conditioned media as intermediate models will be important to dissect how tumor-derived factors shape TTFields-induced TAM skewing.

The polarization of TAMs is a dynamic process influenced by tumor-derived signals, and TAMs often adopt an immunosuppressive M2-like phenotype that supports tumor progression [3,9]. Clinically, high densities of M2 TAMs correlate with poor prognosis, whereas M1-like TAM signatures correlate with better outcomes [8]. This has spurred interest in therapies that “re-educate” TAMs toward an M1 phenotype [2,8]. Our results suggest that TTFields, a non-pharmacological, physical therapy, can accomplish such TAM reprogramming. In our in vitro model, TTFields converted IL-4-polarized macrophages (a proxy for tumor TAMs) to express key co-stimulatory and antigen-presenting molecules and to secrete pro-inflammatory mediators. Notably, TTFields drove phenotype changes akin to those achieved by certain TLR agonists or CD40 agonists used to repolarize TAMs in preclinical studies [9]. Unlike systemic cytokine therapy or small-molecule drugs, TTFields act locally and, being physical fields rather than diffusible agents, are less constrained by biological barriers such as dense tumor stroma or the blood–brain barrier (BBB); our data indicate that they can simultaneously target the tumor and tune the functions of immune cells within the tumor microenvironment.

Mechanistically, our findings highlight a cytoskeleton-linked danger-sensing module that is consistent with, but does not by itself prove, activation of the canonical GEF-H1/RhoA/ROCK axis described in other systems [25]. We provide evidence that TTFields activate GEF-H1 and downstream RhoA-associated signaling in macrophages, leading to phosphorylation of NF-κB p65. Accordingly, we refer to TTFields as activating NF-κB in macrophages, without implying that NF-κB is the sole or exclusive driver of the cytokine program observed. This mechanism aligns with prior work on microtubule-disrupting agents: Kashyap et al. showed that chemotherapeutics like vinblastine activate dendritic cells via GEF-H1 release, which triggers the RhoA-JNK-c-Jun signaling axis and AP-1 transcriptional response [22]. In line with this, Park et al. demonstrated that TTFields treatment of RAW264.7 macrophages elevated levels of pro-inflammatory cytokines (TNF-α, IL-1β, IL-6), nitric oxide (NO), and reactive oxygen species (ROS), and enhanced macrophage-mediated cytotoxicity against tumor cells through NF-κB and p38 MAPK activation [38]. These findings reinforce the concept that TTFields function as a potent activator of innate immune pathways, capable of promoting inflammatory reprogramming of macrophages via cytoskeleton-associated signaling cascades. We extend this concept, demonstrating that physical perturbation of the cytoskeleton can mimic pathogen signals to induce an innate immune response. Further investigation is needed to characterize how TTFields alter actin organization and cellular mechanical properties in macrophages.

While activation of the GEF-H1 can contribute to pro-inflammatory signaling and may support iNOS expression via NF-κB or AP-1 activation, our findings indicate that TTFields-induced iNOS upregulation critically depends on MyD88-dependent signaling, suggesting that TTFields act more as a TLR-like co-signal than through direct cytoskeletal stress alone.

The requirement for MyD88 in TTFields-induced iNOS suggests that TTFields’ effects converge on innate immune pathways typically engaged by Toll-like or interleukin-1 receptors; however, our current data do not identify a specific upstream receptor-ligand pair, and this axis therefore remains to be defined. One plausible interpretation is that TTFields treatment causes macrophages to release endogenous mediators (such as IL-1β) that feed back in an autocrine manner via MyD88-dependent receptors to amplify NF-κB activation. Indeed, microtubule perturbation is known to activate the NLRP3 inflammasome in macrophages, leading to IL-1β/IL-18 release [36], and IL-1/IL-18 signaling could be a key contributor to the iNOS and cytokine induction we observed. An additional, non-mutually exclusive possibility is engagement of MyD88-coupled Toll-like receptors (e.g., TLR2, TLR4) by endogenous danger-associated molecular patterns generated in response to TTFields. Although we did not directly measure inflammasome activation or TLR signaling here, the elimination of iNOS induction by MyD88 blockade strongly implies involvement of one or more MyD88-coupled inflammatory pathways. By contrast, although type I interferon signaling has been implicated in TTFields-mediated immune activation [18], our preliminary analyses did not reveal clear evidence of STING pathway engagement in TTFields-treated macrophages. In essence, TTFields appear to behave as a MyD88-dependent innate immune stimulus for macrophages, much like a damage-associated molecular pattern: by physically stressing microtubules, TTFields can initiate danger signaling cascades inside immune cells, with the precise receptor–ligand combinations remaining to be elucidated. The ability of TTFields to serve as an IFN-γ “co-signal” and induce iNOS is particularly noteworthy. Classically, IFN-γ from T/NK cells licenses macrophages, but full microbicidal/tumoricidal activation requires a second signal, often provided by microbial products [39]. Our data shows that TTFields can substitute for such a second signal (like LPS) to trigger macrophages’ cytotoxic arsenal (iNOS). In tumors, sources of a second signal may be limited (unless infections occur), so TTFields could provide a much-needed inflammatory stimulus to push TAMs over the activation threshold. Interestingly, TTFields induced iNOS to a degree comparable with LPS in our system, suggesting it is a relatively potent stimulus.

Together, these findings support a two-module signaling model in which TTFields engage (i) a cytoskeletal GEF-H1–dependent module that contributes to NF-κB and AP-1 activation and shapes the overall pro-inflammatory tone of macrophages, including surface marker upregulation and cytokine secretion, consistent with prior studies demonstrating GEF-H1-driven RhoA signaling and NF-κB/AP-1 activation following microtubule destabilization in dendritic cells and macrophages; and (ii) a MyD88-dependent receptor module (e.g., IL-1 family receptors or TLRs) that is essential for high-amplitude iNOS induction in IFN-γ-primed macrophages, as evidenced by the complete loss of TTFields-induced iNOS upon MyD88 inhibition but not upon ROCK inhibition. The absence of an inhibitory effect of Y-27632 on iNOS therefore does not contradict this model; rather, it indicates that TTFields-induced iNOS lies predominantly downstream of MyD88-coupled pathways, while RhoA may signal through ROCK-independent effectors (such as mDia or PKN) and/or cooperate with MyD88 pathways in a manner that does not require ROCK for iNOS transcription. Accordingly, we do not propose a strictly linear GEF-H1→RhoA→ROCK→NF-κB→iNOS cascade, but instead a convergent network in which cytoskeletal GEF-H1 signaling and MyD88-dependent receptor signaling operate in parallel and converge on NF-κB/AP-1, with MyD88 providing the critical amplification required for robust iNOS expression. We note that we did not directly inhibit NF-κB or genetically ablate GEF-H1 or MyD88, and therefore our mechanistic conclusions remain correlative. Future studies will be required to validate these pathways more definitively, including direct assessment of RhoA activation in TTFields-treated macrophages, loss-of-function approaches targeting GEF-H1, and evaluation of TTFields responses in MyD88-deficient BMDMs or myeloid-specific conditional knockout models. These experiments, while beyond the scope of the current work, represent important next steps to further dissect the mechanisms underlying TTFields-mediated macrophage activation.

Our findings also carry potential clinical implications. By converting TAMs into a more inflammatory phenotype, TTFields may help overcome one of the major barriers to successful immunotherapy—the immunosuppressive tumor microenvironment. An M1-like TAM population can promote T cell recruitment and function and directly attack tumor cells via iNOS and TNF [3]. In contrast, M2 TAMs contribute to resistance to immunotherapies by dampening T cell responses and fostering immune privilege [8]. Therefore, TTFields’ TAM-reprogramming activity could synergize with immune checkpoint inhibitors and other immunotherapies. Across preclinical NSCLC and other tumor models, TTFields alone have shown limited impact on intratumoral CD8^+^ T cells, whereas protocols in which TTFields are applied alongside immune-checkpoint blockade (anti-PD-1/PD-L1 or anti-CTLA-4) consistently report immunogenic cell death, increased expression of T cell–recruiting chemokines (such as CCL2/8 and CXCL9/CXCL10), higher CD8^+^ TIL numbers, enhanced IFN-γ production, and improved Treg:CD8 ratios, indicating that TTFields can condition the tumor microenvironment to be more permissive for checkpoint inhibitor activity [16,17,40]. Thus, TTFields likely prime the TME, with immunotherapy required to elicit robust T-cell recruitment and activity—consistent with our macrophage findings and supporting combination strategies. Preclinical and clinical evidence indicates that TTFields therapy may increase tumor-infiltrating lymphocytes and synergize with immune checkpoint inhibitors (ICIs) [16,17,19,20,21]. In the phase III LUNAR trial (NCT02973789), in metastatic NSCLC after platinum therapy, subgroup analysis suggested longer overall survival with TTFields and ICIs versus ICIs alone [20]. In the phase III METIS study (NCT02831959) in NSCLC brain metastases, TTFields was associated with a longer time to intracranial and distant intracranial progression, with effects appearing more pronounced among patients receiving ICIs [21]. In newly diagnosed GBM, the phase II 2THE-TOP trial (NCT03430791), TTFields with chemotherapy and ICI after chemoradiation, showed preliminary efficacy, with overall and progression-free survival exceeding historical benchmarks [19]. Our data provide a mechanistic underpinning for such outcomes, as TTFields not only directly impair tumor cells but also activate innate immunity within the tumor. TTFields-induced DAMP release from tumor cells (via immunogenic cell death), together with TAM reprogramming, suggests a two-pronged immunological effect: increased antigen availability and a shift of TAMs toward an antigen-presenting, pro-inflammatory state, thereby counteracting the immunosuppressive TME [16,17]. This raises the possibility that TTFields could convert immunologically “cold” tumors into “hot” ones by recruiting and activating immune cells in situ. Because our immune profiling relied on flow cytometry of dissociated tumors, spatial relationships between TAM phenotypes and ‘hot’/‘cold’ niches could not be assessed; future work using multiplex spatial imaging or spatial transcriptomics will be necessary to determine how TTFields shapes M1/M2 interactions across intratumoral regions and their interplay with immune checkpoint pathways.

In conclusion, this work reveals that TTFields therapy, traditionally viewed purely as a physical anti-mitotic treatment, also has significant immunological activity in polarizing macrophages toward an anti-tumor phenotype. TTFields activated innate immune signaling in macrophages and functionally repolarized them to an M1-like state characterized by enhanced antigen presentation and nitric oxide and cytokine production. By mitigating TAM-mediated immunosuppression, TTFields may create a more favorable environment for immune-mediated tumor clearance. While inflammatory cues can support anti-tumor immunity, chronic or dysregulated inflammation may instead drive immunosuppressive remodeling of the tumor microenvironment [41]. In contrast, the pro-inflammatory activation induced by TTFields may help generate a more immunostimulatory milieu, potentially enhancing responses to immunotherapy as previously demonstrated [16,17,20,40]. Our study therefore provides a scientific rationale for ongoing and future clinical trials integrating TTFields with immune checkpoint inhibitors and other immunomodulators, aiming to translate TAM reprogramming into improved patient outcomes in cancer.

## 4. Materials and Methods

### 4.1. Macrophage Differentiation

Bone marrow cells were harvested from the femurs and tibias of 7-9-week-old female C57BL/6 mice (Harlan Laboratories, Jerusalem, Israel) as previously described [42,43]. Cells were cultured in DMEM medium (Sartorius, Göttingen, Germany; Cat# 01-055-1A) with 10% heat-inactivated FBS (Gibco, Waltham, MA, USA; Cat# A5256701), 1% penicillin/streptomycin (Biowest, Bradenton, FL, USA; Cat# L0022), 2 mM L-Glutamine (Biowest, Bradenton, FL, USA; Cat# X0550), 1 mM sodium pyruvate (Biowest, Bradenton, FL, USA; Cat# L0642), and 10 mM HEPES (Sartorius, Göttingen, Germany; Cat# 03-025-1B), and differentiated into BMDMs by incubation in the presence of 20 ng/mL GM-CSF (Granulocyte-Macrophage Colony Stimulating Factor; Peprotech, Cranbury, NJ, USA; Cat# 315-03) for 7 days. Fresh GM-CSF-containing medium was supplied on day 3. On day 7, adherent macrophages were harvested for polarization experiments.

### 4.2. Macrophage Polarization and TTFields Treatment

BMDMs were polarized to an M1 (classically activated) phenotype by stimulation with 10 ng/mL IFN-γ (Peprotech, Cranbury, NJ, USA; Cat# 315-05) for 24 h, with or without the addition of 10 ng/mL lipopolysaccharide (LPS, InvivoGen, San Diego, CA, USA; Cat# LPS-EB) for 24 h. For M2 (alternatively activated) polarization, BMDMs were stimulated with 100 ng/mL IL-4 (Peprotech, Cranbury, NJ, USA; Cat# 214-14) for 24 h. To examine the effects of TTFields, cells were exposed to TTFields using an in vitro TTFields setup (Inovitro™ system, Novocure, Haifa, Israel) at a field intensity of 1.75 V/cm (root-mean-square) and frequency of 150 kHz. TTFields were applied continuously for 24 h to polarized or unpolarized BMDMs as specified. Control cells (sham exposure) were kept in identical culture conditions without the field. In some experiments, macrophages were IFN-γ-primed and then treated with TTFields for 1 h or 10 ng/mL LPS for 1 h to test whether TTFields can substitute for the classic second signal in M1 activation. Specific inhibitors were added to probe the mechanism of TTFields action: a selective MyD88 inhibitor (10 µM; TJ-M2010-5, MedChemExpress, NJ, USA; Cat# HY-139397) or a Rho-associated kinase (ROCK) inhibitor (15 µM, Y-27632, Cayman Chemical, MI, USA; Cat# 10005583) were added immediately before TTFields exposure and maintained during it. After treatment, cells were harvested for analysis.

### 4.3. Tumor Cell Line

The murine Lewis lung carcinoma cell line LL/2-Luc2 (LL/2; RRID: CVCL_A4CM) was purchased from the American Type Culture Collection (ATCC). Cells were cultured at 37 °C in a humidified incubator containing 5% CO_2_ in DMEM media (Sartorius, Göttingen, Germany; Cat# 01-055-1A) supplemented with 10% (*v*/*v*) FBS (Thermo Fisher Scientific, Waltham, MA, USA; Cat# 2437266), 2 mmol/L L-glutamine (BioWest, Nuaillé, France; Cat# X0550), and penicillin/streptomycin (50 µg/mL, BioWest, Nuaillé, France; Cat# L0022). Cells were used for up to 15 passages after thawing. No authentication test was performed. Cells were tested for Mycoplasma contamination using MycoStrips (InvivoGen, San Diego, CA, USA; Cat# rep-mysnc). The standard laboratory protocol for Mycoplasma testing included screening prior to cell bank cryopreservation, followed by retesting of all thawed cells monthly.

### 4.4. Cytokine Secretion Assays 

Macrophage culture supernatants were collected after 24 h treatments and analyzed using a LEGENDplex™ Mouse Macrophage/Microglia Panel (BioLegend, San Diego, CA, USA; Cat# 740846) to profile secreted factors. The panel quantified 13 mouse cytokines and chemokines relevant to macrophage function: CXCL1 (KC), IL-1β, IL-6, TNF-α, IL-10, IL-12p70, IL-12/23(p40), IL-18, IL-23, CCL17 (TARC), CCL22 (MDC), G-CSF, and active TGF-β1. Assays were performed according to the manufacturer’s protocol and acquired on a Cytek^®^ Northern Lights flow cytometer (Cytek^®^ Biosciences, Fremont, CA, USA). The concentration of each analyte was calculated from standard curves, and values were normalized to the control (untreated) macrophage condition. In some cases, cytokine levels were below detection in M1/M2 control conditions but became detectable after TTFields; these were noted qualitatively.

### 4.5. Western Blot

To examine signaling pathway activation, cell lysates were prepared from BMDMs at various time points during TTFields exposure (0, 0.25, 0.5 and 1 h). Protein extracts (20–30 µg per lane) were resolved by SDS-PAGE and transferred to PVDF membranes. Blots were probed with primary antibodies (see Table 1) against total and phosphorylated forms of GEF-H1 (Ser886), NF-κB (p65, Ser536), and c-Jun (Ser63), followed by HRP-conjugated secondary anti-rabbit or anti-mouse antibodies (Abcam, Cambridge, UK; Cat# AB6721 or Cat# AB97023, respectively). GAPDH was used as a loading control. Bands were visualized by Immobilon Forte Western HRP substrate (Millipore, Burlington, MA, USA; Cat# WBLUF) and quantified by densitometry. The intensity of phosphorylated protein bands was normalized to total protein and to GAPDH, then expressed relative to the value in untreated cells at time 0.

### 4.6. Animal Experimentation Design

Experimental procedures involving animal housing, anesthesia, and Tumor Treating Fields (TTFields) application were conducted in accordance with previously established protocols [26,44]. Male C57Bl/6 mice (10–12 weeks old; Harlan Laboratories, Jerusalem, Israel) were orthotopically implanted with 3 × 10^3^ LL/2 cells suspended in 5 µL of growth factor-reduced Matrigel (1:1 dilution; Corning, Corning, NY, USA; Cat# 354263) via direct intrapulmonary injection. Six days post-inoculation, magnetic resonance imaging (MRI) was used to measure tumor size and verify its location. Only animals demonstrating confined tumor growth were subsequently randomized to one of two treatment groups, (1) sham control or (2) TTFields-treated group (1.86 ± 0.67 V/cm RMS, 150 kHz), using the Inovivo™ delivery system (Novocure, Haifa, Israel). On day 7 following tumor inoculation, TTFields therapy was initiated continuously for 10 consecutive days, following protocols designed to replicate clinical treatment regimens [44]. This 10-day treatment window was selected to ensure sufficient and sustained TTFields exposure to elicit measurable immune effects, consistent with previous preclinical TTFields studies evaluating immunological endpoints [17]. Device usage was monitored to ensure compliance with treatment exposure (≥18 h/day), in alignment with clinical guidelines for optimal therapeutic effect. Sham-treated mice were fitted with non-functional arrays identically shaped and sized that generated equivalent thermal conditions (~38.5 °C) and were positioned over the thoracic region in an orientation matching that of the functional arrays.

### 4.7. Magnetic Resonance Imaging (MRI) Acquisition

Tumor imaging was performed using the ICON 1 Tesla MRI system (Bruker Biospin, Ettlingen, Germany). Mice were anesthetized and placed in a prone position inside a dedicated body coil. Transverse T2 weighted RARE images were acquired using the following parameters: repetition time—2000 ms; excitation time—56 ms; RARE factor—12; number of scans—8; number of slices—12; slice thickness—0.9 mm; matrix size—128 × 128; FOV—28 mm; in-plane resolution—0.22 × 0.22 mm; acquisition time—3 min 12 s.

### 4.8. Sample Collection and Tissue Preparation

At endpoint, mice were euthanized, and tumors were harvested. Tumor dimensions were recorded, and then processed using enzymatic dissociation into single-cell suspensions utilizing a gentleMACS™ dissociator (Miltenyi Biotec, Bergisch Gladbach, Germany) and tumor dissociation kit (Miltenyi Biotec, Bergisch Gladbach, Germany; Cat# 130-096-730) according to the manufacturer’s instructions. Single-cell suspensions were subsequently stained for further analysis.

### 4.9. Flow Cytometry Analysis

Following in vitro treatments, macrophages were collected and stained for surface markers to assess polarization and activation status. Cells were labeled with fluorochrome-conjugated antibodies against F4/80 (pan-macrophage marker, APC/Fire810, BioLegend, San Diego, CA, USA; Cat# 123166), MHC class II (I-A/I-E, BV510, BioLegend, San Diego, CA, USA, Cat# 107635), and CD80 (B7-1, PE, BioLegend, San Diego, CA, USA; Cat# 104708) for M1 activation, and CD206 (mannose receptor, BV711, BioLegend, San Diego, CA, USA; Cat # 141727) for M2 phenotype. For intracellular markers, cells were fixed and permeabilized using the Cyto-Fast™ Fix/Perm Buffer Set (BioLegend, San Diego, CA, USA; Cat# 426803) and stained for inducible nitric oxide synthase (iNOS, AF488, Invitrogen, Waltham, Massachusetts, USA; Cat# 53-5920-82) as an M1 functional enzyme, and Arg-1 (APC (Invitrogen, Waltham, Massachusetts, USA; Cat# 17-3697-82) as an M2 marker. Dead cells were excluded by staining with Zombie NIR Fixable Viability dye (BioLegend, San Diego, CA, USA; Cat# 423106) before fixation.

Data were acquired on Cytek^®^ Northern Lights flow cytometer (Cytek^®^ Biosciences, Fremont, CA, USA) and analyzed using FlowJo software (Becton Dickinson, Ashland, OR, USA). Populations were gated on singlets and F4/80+ macrophages, and results were reported as percentage of positive cells or median fluorescence intensity (MFI) for each marker.

For analysis of TAMs in single-cell suspensions from LL/2 tumors, Viobility 405/452 Fixable Dye (Miltenyi Biotec, Bergisch Gladbach, Germany, Cat# 130-130-420) was used for the discrimination of dead cells. Mouse Fc block (anti-CD16/CD32 (clone 93), Biolegend, San Diego, CA, USA; Cat# 156604) was used prior to staining with the fluorochrome-conjugated anti-mouse antibodies listed in Table 2. For staining of intracellular lineage-defining factors, Cyto-Fast™ Fix/Perm Buffer Set (Biolegend, San Diego, CA, USA; Cat# 426803) was utilized according to manufacturer instructions. The gating strategy is outlined in Figure S3.

### 4.10. Statistical Analysis

Experiments were performed on at least three independent biological replicates. Data are presented as mean ± standard error. Statistical significance was calculated using GraphPad Prism 10 software (La Jolla, San Diego, CA, USA), with the specific tests used mentioned in figure legends. Differences were considered to be statistically significant for *p* values of ≤0.05 and were indicated as * *p* < 0.05; ** *p* < 0.01; *** *p* < 0.001.

## Figures and Tables

**Table 1:** List of antibodies for immunoblotting.

Antigen	Dilution	Cat #	Manufacturer
NF-κB	1:1000	8242	Cell signaling *
phosphorylated NF-κB (Ser536)	1:1000	3033	Cell signaling *
c-Jun	1:1000	9165	Cell signaling *
phosphorylated c-Jun (Ser63)	1:500	9261	Cell signaling *
GEF-H1	1:1000	4076	Cell signaling *
phosphorylated GEF-H1 (Ser886)	1:1000	14143	Cell signaling *
GAPDH	1:2500	32233	Santa Cruz **

* Cell Signaling, Danvers, MA, USA. ** Santa Cruz, Dallas, TX, USA.

**Table 2:** List of antibodies for TAMs staining in single-cell suspensions.

Antigen	Fluorochrome	Cat #	Manufacturer
F4/80	PE	123110	BioLegend *
CD11b	PerCP	101230	BioLegend *
CD45	VioGreen	130-110-665	Miltenyi Biotec **
Arginase-1	APC	17-3697-82	Invitrogen ***
CD206	PerCP Cy5.5	141716	BioLegend *
iNOS	AF488	53-5920-82	Invitrogen ***

* BioLegend, San Diego, CA, USA. ** Miltenyi Biotec, Bergisch Gladbach, Germany. *** Invitrogen, Waltham, Massachusetts, USA.

## Data Availability

All data generated or analyzed during this study are included in this article. Further inquiries can be directed to the corresponding authors.

## Supplementary Materials

The following supporting information can be downloaded at: https://www.mdpi.com/article/10.3390/ijms262412086/s1.

## Abbreviations

The following abbreviations are used in this manuscript:

AF488	Alexa Fluor 488
AIM2	Absent in Melanoma 2
ANOVA	Analysis of variance
AP-1	Activator protein-1
APC (cells)	Antigen-presenting cell(s)
APC (fluor)	Allophycocyanin (fluorescent dye)
ATCC	American Type Culture Collection
BBB	Blood–brain barrier
BMDMs	Bone marrow–derived macrophages
BV510/BV711	Brilliant Violet 510/711 (fluorochromes)
cGAS	cyclic GMP–AMP synthase
CD11b	Cluster of Differentiation 11b
CD45	Cluster of Differentiation 45
CD80	Cluster of Differentiation 80
CD206	Cluster of Differentiation 206 (mannose receptor)
CO_2_	Carbon dioxide
CXCL1 (KC)	Chemokine (C-X-C motif) ligand 1 (Keratinocyte-derived chemokine)
DAMPs	Damage-associated molecular patterns
DMEM	Dulbecco’s Modified Eagle Medium
FBS	Fetal bovine serum
G-CSF	Granulocyte colony-stimulating factor
GEF-H1	Guanine nucleotide exchange factor-H1 (ARHGEF2)
GAPDH	Glyceraldehyde-3-phosphate dehydrogenase
GBM	Glioblastoma
GM-CSF	Granulocyte-macrophage colony-stimulating factor
HEPES	4-(2-hydroxyethyl)-1-piperazineethanesulfonic acid
HMGB1	High mobility group box 1
HRP	Horseradish peroxidase
ICD	Immunogenic cell death
ICI/ICIs	Immune checkpoint inhibitor(s)
IFN-γ	Interferon gamma
IL-1β	Interleukin-1 beta
IL-6	Interleukin-6
IL-10	Interleukin-10
IL-12p70	Interleukin-12 p70
IL-12/23 (p40)	Interleukin-12/23 p40 subunit
IL-18	Interleukin-18
IL-23	Interleukin-23
iNOS	Inducible nitric oxide synthase
IκB	Inhibitor of kappa B
JNK	c-Jun N-terminal kinase
LPS	Lipopolysaccharide
MAPK	Mitogen-activated protein kinase
MDC (CCL22)	Macrophage-derived chemokine (Chemokine [C-C motif] ligand 22)
MFI	Median fluorescence intensity
MHC-II	Major histocompatibility complex class II
MRI	Magnetic resonance imaging
MyD88	Myeloid differentiation primary response 88
NF-κB	Nuclear factor kappa B
NLRP3	NOD-, LRR- and pyrin domain-containing protein 3 (inflammasome)
NSCLC	Non-small cell lung cancer
PD-1	Programmed cell death protein 1
PD-L1	Programmed death-ligand 1
PE	Phycoerythrin (fluorochrome)
PerCP/PerCP-Cy5.5	Peridinin-chlorophyll-protein/tandem dye
PVDF	Polyvinylidene difluoride
RMS	Root mean square
ROCK	Rho-associated kinase
RhoA	Ras homolog family member A
ROS	Reactive oxygen species
SDS-PAGE	Sodium dodecyl sulfate–polyacrylamide gel electrophoresis
SD	Standard deviation
SEM	Standard error of the mean
STING	Stimulator of Interferon Genes
TAM(s)	Tumor-associated macrophage(s)
TARC (CCL17)	Thymus and activation-regulated chemokine (Chemokine [C-C motif] ligand 17)
TGF-β/TGF-β1	Transforming growth factor beta/beta 1
TIL(s)	Tumor-infiltrating lymphocyte(s)
TLR	Toll-like receptor
TNF-α	Tumor necrosis factor alpha
TTFields	Tumor Treating Fields
VEGF	Vascular endothelial growth factor
VioGreen	Fluorochrome (Miltenyi)
