# Supplementary material for: Pro-Inflammatory Macrophage Phenotype Skewing Induced by Tumor Treating Fields (TTFields)
Source: Int J Mol Sci. 2025 Dec 16;26(24):12086. doi: 10.3390/ijms262412086 (PMC12733770; doi:10.3390/ijms262412086)
Supplement: Supplementary file 1 [file ijms-26-12086-s001.zip › ijms-4014722-supplementary.pdf]

Figure S1. Cytokine and chemokine secretion profile measured by multiplex analysis in supernatants BMDMs.

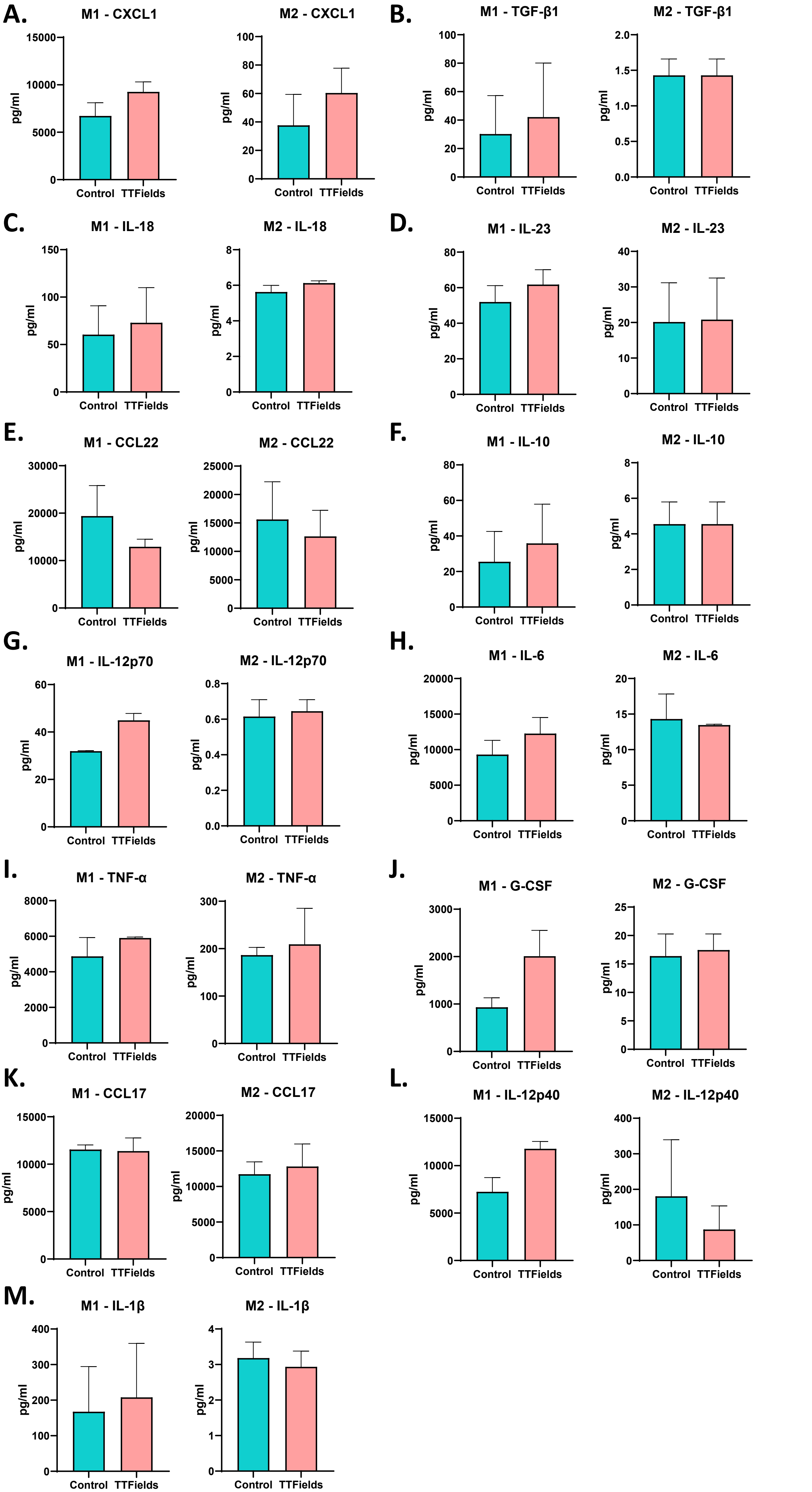

Figure S2. Cytokine and chemokine secretion profile measured by multiplex analysis in supernatants from IFN-γ–primed BMDMs.

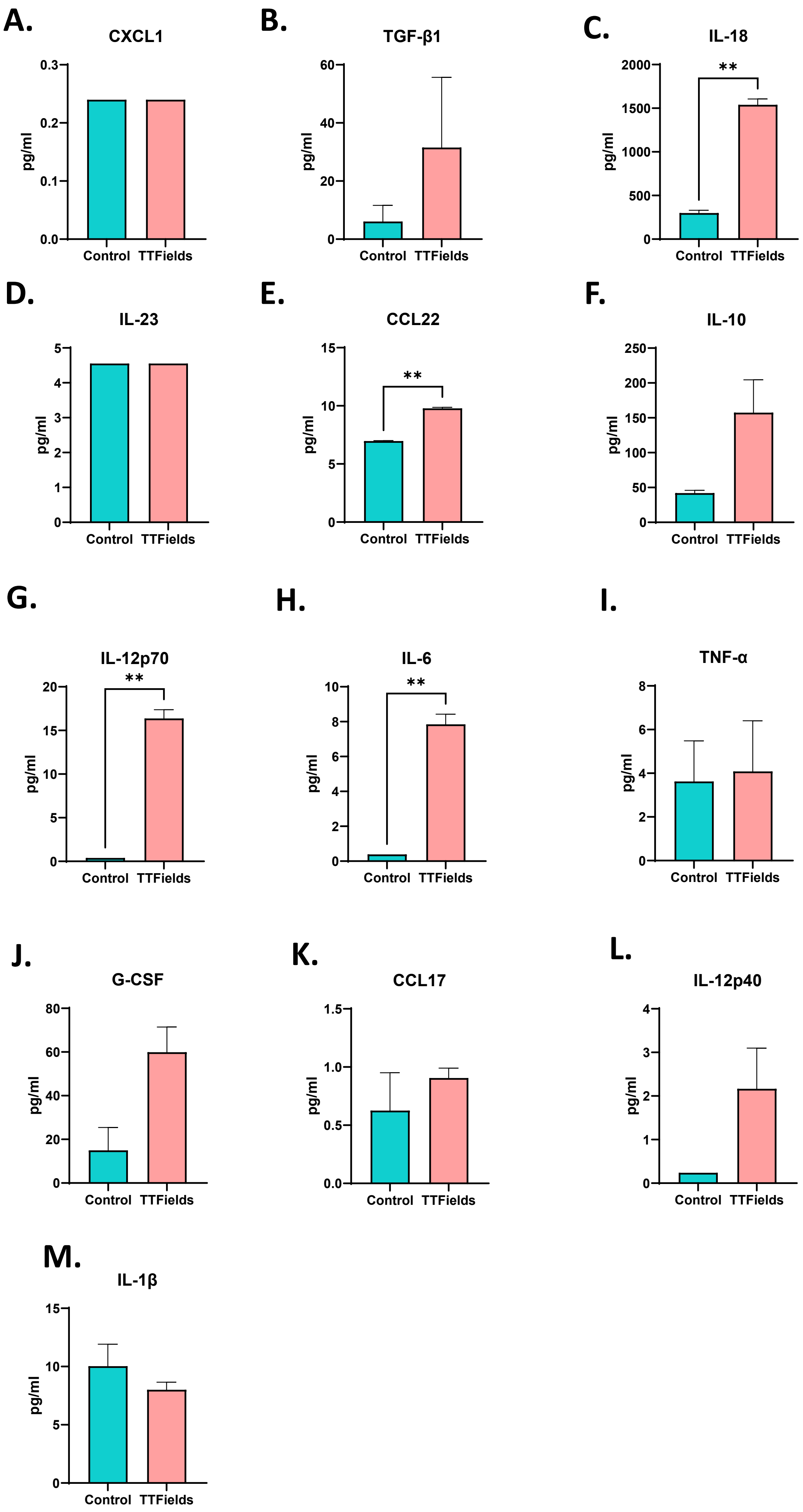

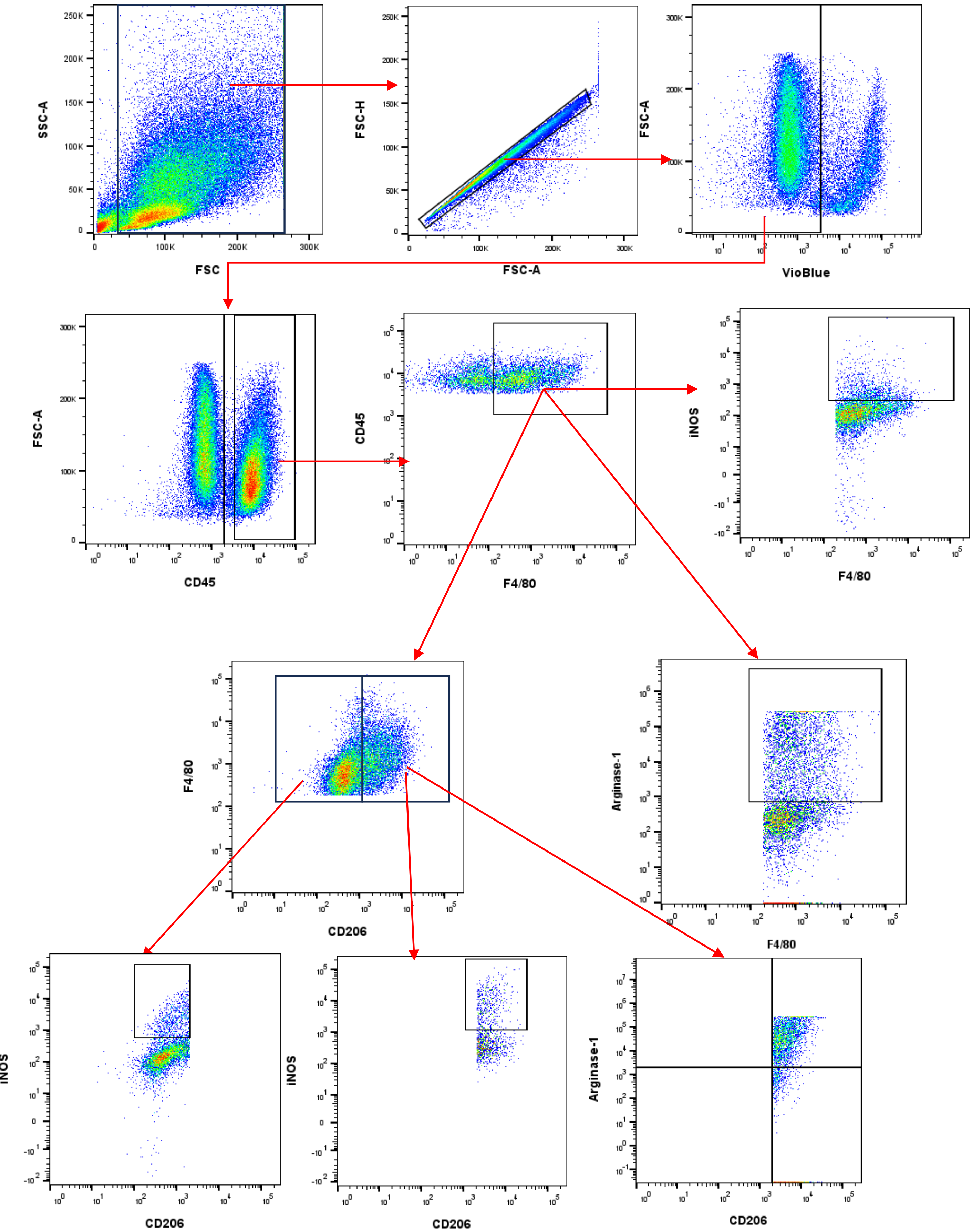

Figure S3. Flow cytometric gating strategy for discerning innate immune subsets

Leukocytes were first identified and gated as **CD45$^+$** cells. Within the CD45$^+$ population, subsets were further defined as follows:

Macrophages (total): CD45$^+$F4/80$^+$

M1-like macrophages: CD45$^+$F4/80$^+$CD206$^-$iNOS$^+$

M2-like macrophages: CD45$^+$F4/80$^+$CD206$^+$Arg1$^+$
